# Comparative analysis of lumen enlargement mechanisms achieved with the bifurcation dedicated BiOSS^®^ stent versus classical coronary stent implantations by means of provisional side branch stenting strategy: an intravascular ultrasound study

**DOI:** 10.1007/s10554-013-0264-0

**Published:** 2013-07-19

**Authors:** Robert J. Gil, Jacek Bil, Aleksandra Michałek, Dobrin Vassiliev, Ricardo A. Costa

**Affiliations:** 1 Department of Invasive Cardiology, Central Hospital of the Internal Affairs Ministry, 137 Woloska Street, 02-507 Warsaw, Poland; 2 Institute of Experimental and Clinical Medicine, Polish Academy of Science, Warsaw, Poland; 3 National Heart Hospital, Sofia, Bulgaria; 4 Instituto Dante Pazzanese de Cardiologia, Sao Paulo, Brazil

**Keywords:** Coronary bifurcation lesions, Dedicated bifurcation devices, Intravascular ultrasound, Percutaneous coronary intervention

## Abstract

The aim of this study was to analyze the mechanisms of lumen enlargement in bifurcation lesions, as assessed by intravascular ultrasound (IVUS), after percutaneous treatment with classic provisional “T” stenting with conventional drug-eluting stents (DES) versus bifurcation dedicated BiOSS^®^ (Balton, Warsaw, Poland) stent. In this prospective study between Jan and Dec/11, 32 patients with single de novo coronary bifurcation lesions suitable for treatment with BiOSS stents were randomized (1:1). IVUS method included pre- and post-procedure analysis in the parent vessel. Vessel, lumen and plaque cross-sectional areas were determined at the target lesion [minimum lumen area (MLA) site], proximal limb, distal limb, and “window”—defined as the segment between the carina (flow divider) and the vessel wall at the level of the side branch inflow. All lesions were treated with provisional approach and only 1 case in BiOSS group had a stent implanted in the side branch. Angiographic and IVUS results including MLA at the target site and proximal/distal references were similar. However, mean window length—largest diameter within the window, was similar at baseline, but BiOSS measured significantly longer at postprocedure (2.21 ± 0.37 vs. 1.76 ± 0.52 mm, *p* = 0.01). In addition, the magnitude of changes in vessel (27 ± 24 % vs. 9 ± 10 %, *p* = 0.01) and plaque (2 ± 26 % vs. −2 ± 26 %, *p* = 0.02) areas at the window were significantly different for DES versus BiOSS groups, respectively. The contribution of vessel extension for lumen enlargement represented 54 versus 43 %, 130 versus 46 %, 98 versus 80 % and 51 versus 19 % of the result achieved at the proximal limb, window, distal limb and MLA sites for DES versus BiOSS, respectively; as for plaque re-distribution, results were 36 versus 57 %, −30 versus 54 %, 2 versus 20 %, and 49 versus 81 %, at the proximal limb, window, distal limb and MLA sites, respectively. These results suggest different mechanisms of lumen enlargement comparing conventional DES versus BiOSS dedicated bifurcation stent, which can impact side branch compromise during procedure.

## Introduction

Recently published data on BiOSS^®^ Expert stent (Balton, Warsaw, Poland) Registry showed that this bifurcation dedicated device is very promising both for the operator as user`s friendly and for patients in regard of good immediate and short-term clinical results [1]. This balloon-expandable stent is made of 316L stainless steel and has unique construction that consists of two parts with different diameters connected with two struts. Its delivery system is based on a bottle shaped balloon (Bottle^®^, Balton, Poland) which restores “proximal” main vessel and “distal” main branch sizes without the need of an additional dilatation called kissing ballooning [1]. As it was proven, the construction of the BiOSS stent prevents from carina displacement—the basic mechanism of side branch compromise during bifurcation percutaneous coronary intervention [2]. This step-up mid zone of the BiOSS stent created by two relatively short connecting struts (mean length 1.2 mm) on the one hand should secure good access to SB and stent`s flexibility, but on the other may be its “weak point” due to lower radial forces and a low dose of an antiproliferative substance. Theoretically, this part of the BiOSS stent may be responsible for not so optimal results achieved immediately after its implantation and during late follow-up.

The aim of the study was to analyze the mechanisms of lumen enlargement after the coronary bifurcation dedicated stent BiOSS versus the classical stent implantation according to the provisional “T” stenting strategy, as determined by intravascular ultrasound (IVUS) measurements.

## Methods

### Study population

Between January 2011 and December 2011, a total of 32 patients with stable coronary artery disease were consecutively enrolled and randomized in this prospective study at a single center. The main inclusion criteria were presence of de novo coronary bifurcation lesion suitable for treatment with the BiOSS device, serum creatinine level below 2.0 mg/dL and the ability to take dual antiplatelet therapy for 12 months. The main exclusion criteria were ST-elevation acute coronary syndrome and the lack of signed informed consent. Overall, patients had to be qualified by the institution’s Heart Team for percutaneous revascularization. However due to BiOSS stent size availability in that time (maximal diameter 3.75 mm) patients with so called big left main stems (proximal reference diameter ≥4.0 mm by QCA) were excluded. The studied population was divided in two groups, both consisting of 16 patients (randomization 1:1), according to the device and strategy used for bifurcation treatment: drug-eluting stents (DES) with PTS strategy (DES group) versus BiOSS Expert dedicated DES (BiOSS group).

The study was conducted according to the principles of the Declaration of Helsinki, and the protocol was approved by the institutional review board. Written informed consent was obtained from all patients before procedure.

### Procedure

All procedures were performed in a standard way via the radial or femoral access using guiding catheters of 6- and 7-Fr. in diameter. A single stent implantation in the main vessel + main branch across side branch was the default strategy in all patients. A stent in side branch was implanted only in case of ostial residual stenosis greater than 70 % after balloon dilatation and/or significant flow impairment after main vessel + main branch stenting and/or flow limiting dissection. The recommended strategy was to choose the stent diameter according to distal reference diameter (localized in main branch). Overall, the following consecutive steps for the implantation protocol were considered:

1. wiring of both branches;
2. main vessel predilatation and/or side branch predilatation according to the operator’s decision;
3. stent implantation—balloon inflation at 10–12 atm for at least 20 s;
4. stent postdilatation with Bottle balloon at operator’s discretion in BiOSS group, and with non-compliant balloon in DES group (separately for main vessel and main branch);
5. side branch postdilatation if presence of side branch ostial stenosis >70 %;
6. final kissing balloon inflation at operator’s discretion.

In regard to the antithrombotic therapy, all patients were pre-treated with conventional loading doses of aspirin and clopidogrel (300–600 mg) at least 48 h before procedure, followed by aspirin 150 mg/day and clopidogrel 75 mg/day. Use of glycoprotein IIb/IIIa inhibitors was left to the operator’s discretion. At postprocedure, dual antiplatelet therapy including aspirin 75–150 mg/day plus clopidogrel 75 mg/day was prescribed for 12 months. After insertion of the arterial sheath, each patient received unfractionated heparin (70–100 IU/kg); additional bolus was given to maintain an activated clotting time >200 s.

Patients were clinically evaluated (medical evolution, physical examination, ECG) at 30 days, 6, 9 and 12 months. Angiographic re-evaluation was planned at 12 month.

### Angiographic analysis

All coronary angiograms were recorded after an intracoronary administration of 200 μg of nitroglycerin. Two orthogonal views were chosen to visualize the target lesion. A quantitative angiographic analysis was performed using commercially available software (QCA-CMS version 5.0, Medis, Leiden, the Netherlands). Catheter calibration was used in all cases. The main vessel (arterial segment before side branch take-off), the main branch (arterial segment beyond the side branch ostium), and the side branch were individually analyzed [3]. Thus, the following parameters were determined: reference vessel diameter, minimal lumen diameter (MLD) and percent diameter stenosis for the main vessel, main branch, and side branch before and after stent implantation. All reference diameters (user-defined) were measured within 5 mm from the end of the angiographically visible plaque or stenosis in all 3 segments of the bifurcation. Percent diameter stenosis (for each segment) was calculated by the following formula: diameter stenosis = [1 − (MLD/reference vessel diameter)] × 100. Measurement of α angle (carina angle) was performed as previously described [4, 5]. Angiographic success was defined by main vessel and main branch diameter stenosis less than 20 % and side branch ostial stenosis less than 70 % without significant dissection and flow impairment [6].

### Intravascular ultrasound

After baseline coronary angiography, an IVUS catheter (Eagle Eye Gold, Volcano Corporation, Rancho Cordova, CA, USA) was advanced distally to the bifurcation to be stented. Pullback was performed at the speed of 0.5 mm/s. until the guiding catheter was reached. Plaques were characterized by their appearance on IVUS images (soft, hard, mixed) according to widely accepted definitions [7]. External elastic membrane (EEM) was taken as a border of vessel’s total cross-sectional area (CSA) (vessel area, VA) and was identified as the edge between hypoechoic media and hyperechoic adventitia. Lumen area (LA) was measured by tracing the leading edge of the intima before stenting and of stent after intervention. Plaque plus media CSA was accepted as a surrogate for plaque area (PLA) because by IVUS was not possible to separate media from plaque. Each of these parameters and both references (proximal and distal) were analyzed in single slices. Plaque burden (PB) was calculated according to the formula: (VA − LA)/VA. The reference segments were the least disease IVUS CSA (largest lumen with smallest plaque + media) ≤ 2–3 mm distal and proximal to the stented segment [7]. EEM, lumen and plaque through the entire lesion (stent + 2–3 mm from the stent`s edge) were measured at 1 mm intervals. Overall, EEM, lumen and plaque volume were calculated based on the Simpson’s rule.

IVUS was performed in all cases in the parent vessel (main vessel + main branch) before and after stenting. The following IVUS measurements were performed within the bifurcation anatomy during analysis:

- Minimal lumen area (MLA), VA and corresponding PLA at the lesion site (main vessel or main branch);
- MLA, VA and PLA at the level of the proximal rim of the side branch ostium (also named proximal limb);
- MLA, VA and PLA at the level of distal rim of the side branch ostium (also named distal limb);
- Window length, defined as the largest diameter between carina and vessel wall (or between stent struts) at the level of side branch inflow as seen from the main vessel;
- Window, defined as the CSA of the segment between proximal and distal limb at the level of side branch inflow
- MLA, VA and PLA measured at the level of the most diseased segment within the window length (in-bifurcation segment);
- Plaque volume (PV) at baseline, defined as the volumetric reconstruction of the lesion located between proximal and distal references;
- Volumetric analysis:
  - Plaque Volume (PV) at the bifurcation, defined as the PV of vessel segment along the in-bifurcation segment including CSAs at proximal limb, window length and distal limb;
  - PB at the bifurcation, defined as the vessel segment along the in-bifurcation segment including CSAs of proximal limb, window length and distal limb.
  - The magnitude of changes in CSA in % at regions of interest was determined by the following formula: [(CSA at preprocedure—CSA at postprocedure)/CSA at preprocedure] × 100.

**Fig. 1:**
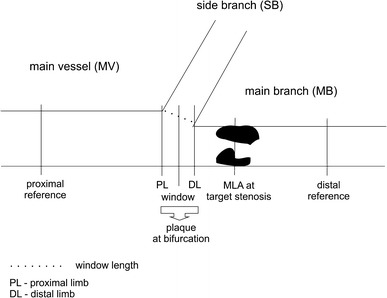
Schematic illustration of the bifurcation anatomy highlighting the regions of interest assessed by IVUS analysis. *MLA* minimum lumen area

Figure 1 illustrates sites for quantitative IVUS measurements. *Off*-*line* quantitative IVUS analysis was performed by two independent investigators (A. M, J. B), unaware of the QCA measurements.

### Statistical analysis

Continuous variables are presented as means ± one standard deviation. Categorical variables are expressed as percentages. The differences between groups were examined with paired or unpaired t-tests as appropriate, with normal distributions. Comparison among groups for categorical variables was made with the Chi square method. All statistical analyses were performed using SPSS version 13.0 for Windows (SPSS Inc., Chicago, Illinois). A *p* value <0.05 was considered significant.

## Results

There were 32 patients (75 % male) with stable coronary artery disease enrolled. Overall, most baseline characteristics did not differ among groups apart from diabetes, which was significantly more frequent in the BiOSS group, and smoking, which on the contrary, was more frequent in the DES group. Only in the BiOSS group (8 cases), an unprotected left main was the target vessel. The left anterior descending artery was dominantly affected in the DES group (81.3 %) versus 25 % in the BiOSS group (25 %). In addition, there were no significant differences in regard to bifurcation types according to the Medina classification. Table 1 depicts baseline clinical and angiographic characteristics.

**Table 1:** Baseline clinical and angiographic characteristics

Variable	Group 1 (DES)	Group 2 (BiOSS)
n	16	16
Age, years	64 ± 11	70 ± 9
Male gender, n (%)	11 (70.8)	13 (81.3)
Diabetes, n (%)	3 (18.8)	6 (37.5)*
Hypertension, n (%)	14 (87.5)	12 (75.0)
Dyslipidemia, n (%)	6 (37.5)	4 (25.0)
Smoking history, n (%)	6 (37.5)	1 (6.3)*
Previous MI, n (%)	7 (43.8)	7 (43.8)
Previous PCI, n (%)	10 (62.5)	10 (62.5)
Previous CABG, n (%)	0	1 (6.3)
Clinical presentation, n (%)		
Stable angina	16 (100.0)	16 (100.0)
Target vessel, n (%)		
LAD	13 (81.3)	4 (25.0)*
LCx	2 (12.5)	4 (25.0)
RCA	1 (6.3)	0 (0.0)
LM	0 (0.0)	8 (50.0)*
Medina classification, n (%)		
1,1,1	6 (37.5)	8 (50.0)
0,1,1	2 (12.5)	3 (18.8)
1,0,1	3 (18.8)	3 (18.8)
1,1,0	4 (25.0)	2 (12.5)
1,0,0	1 (6.3)	0 (0.0)
0,1,0	0 (0.0)	0 (0.0)
0,0,1	0 (0.0)	0 (0.0)

Values are presented as mean ± standard deviation or frequencies (percent of the total)

*CABG* coronary artery bypass graft surgery, *LAD* left anterior descending, *LCx* left circumflex, *LMS* left main (unprotected), *MI* myocardial infarction, *PCI* percutaneous coronary intervention, *RCA* right coronary artery

* *p* < 0.05 compared to DES group

### Procedural and QCA results

Procedural data is shown in Table 2. During procedure, patients enrolled in the DES group were treated with the following stents: Luc-Chopin^2^ (Balton, Warsaw, Poland) in 8 cases, Promus (Boston Scientific, Natick, MA, USA) in 4 cases, and Xience V (Abbott Vascular, Santa Clara, CA, USA) in 4 cases. As for the BiOSS group, the BiOSS dedicated device was successfully implanted in all cases. The main branch was predilated in the majority of cases in both groups (>80 %) and final kissing balloon inflation was 62.5 % in DES versus 50 % in BiOSS groups. Device success rate was 100 %, but there was the necessity to implant an additional stent only in 1 patient in the BiOSS group due to the significant side branch dissection after predilatation. QCA data is presented in the Table 3. A postprocedure, side branch ostial residual stenosis was 46 % in the DES group versus 32 % in the BiOSS group (*p* < 0.04). Moreover, the alpha (α) angle was significantly higher in the BiOSS group (probably to the high contribution at the left main lesions).

**Table 2:** Procedural data

Variable	DES	BiOSS
n	16	16
Vascular access, n (%)		
Femoral	2 (12.5)	4 (25.0)
Radial	14 (87.5)	12 (75.0)
Guiding-catheter size, n (%)		
6-Fr.	16 (100.0)	4 (25.0)*
7-Fr.	0 (0.0)	12 (75.0)*
Predilatation, n (%)		
MV + MB	13 (81.3)	14 (87.5)
SB	9 (56.3)	12 (75.0)
Both branches	7 (43.8)	9 (56.3)
Study stent implanted^a^	16 (100.0)	16 (100.0)
Nominal stent length, mm	18.94 ± 6.14	16.13 ± 1.5
Nomimal stent diameter, mm		
MV	3.41 ± 0.36	3.66 ± 0.27
MB	–	3.01 ± 0.18
Additional stent implanted, n (%)		
MV + MB	0 (0.0)	0 (0.0)
SB	0 (0.0)	1 (6.3)
Balloon postdilatation, n (%)		
MV + MB (Bottle balloon)	0 (0.0)	3 (18.8)
SB	12 (75.0)	7 (43.8)
Final KBI	10 (62.5)	8 (50.0)
Contrast volume, ml	195 ± 71	171 ± 38
Fluoroscopic time, min	14.2 ± 6.4	17.5 ± 8.5
Procedural time, min	83 ± 27	74 ± 25

Values are presented as frequencies (percent of the total) or mean ± standard deviation

*KBI* kissing-balloon inflation, *MB* main branch, *MV* main vessel, *SB* side branch

* *p* < 0.05 versus DES group

^a^According to randomization

**Table 3:** Baseline and final QCA

Variable	DES (n = 16)		BiOSS (n = 16)		*p* value	
	Pre (a)	Post (b)	Pre (c)	Post (d)	Pre (a vs. c)	Post (b vs. d)
Lesion length, mm						
MV + MB	17.9 ± 4.0	–	15.3 ± 5.0	–	0.06	–
MB only	9.8 ± 7.9	–	6.0 ± 4.2	–	0.10	–
SB	5.0 ± 4.4	–	4.9 ± 6.4	–	0.97	–
RVD, mm						
MV	3.4 ± 0.4	3.4 ± 0.4	3.4 ± 0.4	3.5 ± 0.4	0.67	0.29
MB	2.7 ± 0.5	2.8 ± 0.5	2.9 ± 0.5	2.8 ± 0.3	0.39	0.84
SB	2.2 ± 0.4	2.2 ± 0.5	2.4 ± 0.6	2.4 ± 0.5	0.18	0.25
% DS						
MV	51 ± 18	3 ± 10	52 ± 19	8 ± 12	0.88	0.26
MB	50 ± 18	2 ± 19	52 ± 16	3 ± 11	0.74	0.77
SB	46 ± 11	46 ± 17	38 ± 15	32 ± 20	0.09	0.04
α angle, degress	42.0 ± 13.5	40.4 ± 8.8	52.1 ± 22.0	53.5 ± 20.0	0.15	0.04

Values are presented as mean ± standard deviation

*DS* diameter stenosis, *MB* main branch, *MV* main vessel, *RVD* reference vessel diameter, *SB* side branch

### IVUS results

**Fig. 2:**
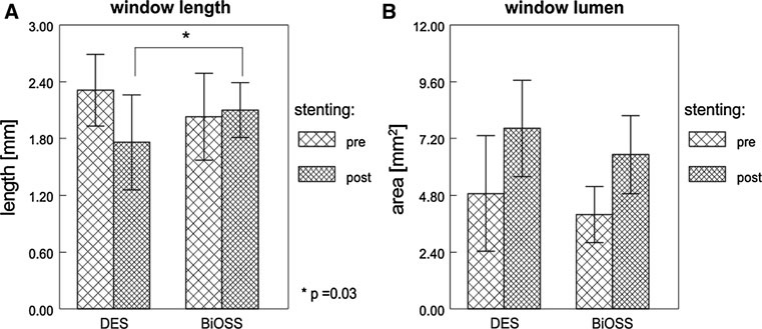
Changes in window length (**a**) and lumen CSA at window (**b**) comparing pre- versus postprocedure measurements within each group. *p < 0.05. *CSA* cross-sectional area

**Fig. 3:**
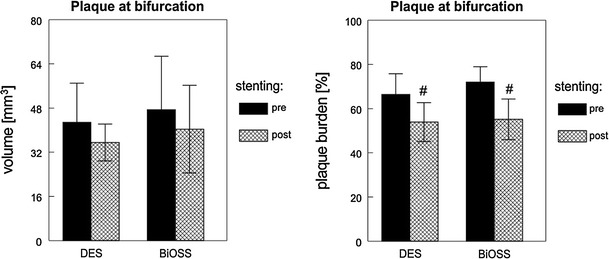
IVUS volumetric analysis comparing pre- and postprocedure measurements within each group showing changes in PV (*left*) and PB (*right*) at in-bifurcation segment

**Fig. 4:**
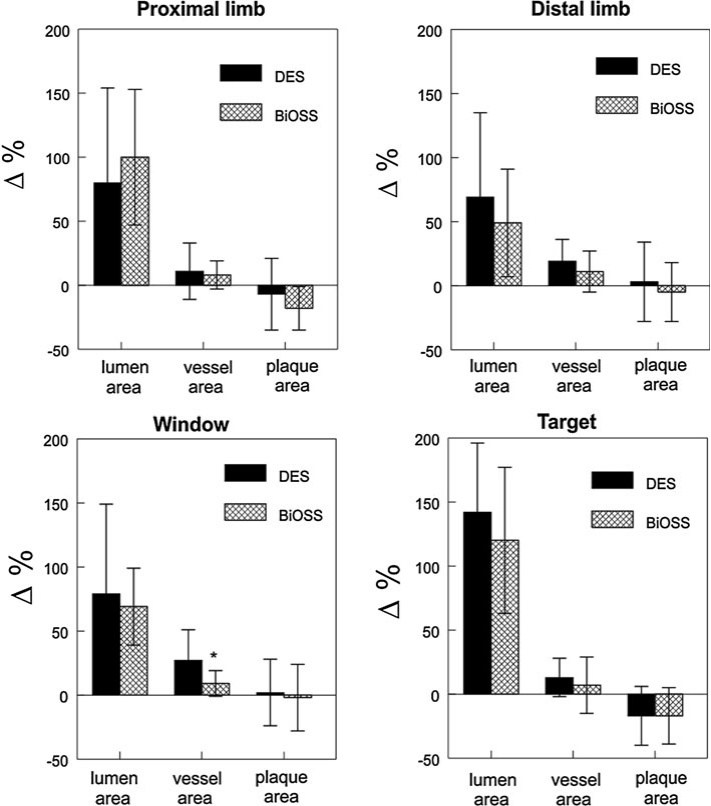
Lumen, vessel and plaque CSA variations (%) at regions of interest

Plaque characteristics analysis showed even distribution for individual plaque types including soft, hard and mixed types in 23.5, 32.4 and 44.1 % in DES versus 28.9, 36.8 and 34.2 % in BiOSS, respectively. Similarly, no significant differences were found regarding lesion length (as assessed by IVUS) and other quantitative parameters at both proximal and distal references. Pre- and postprocedural measurements are presented in the Table 4. It is important to underline that, excluding preprocedural PLA for proximal limb (*p* = 0.05), the parameters before stenting did not differ significantly between both groups. Overall, the successful stent implantation caused significant increase in LA at the target stenosis (MLA site), proximal limb, distal limb and window within each group, but they did not differ significantly between them, i.e., DES versus BiOSS, Table 4. Actually, the only significant difference between DES versus BiOSS after intervention was found for window length, which was significantly longer in the group where the BiOSS stent was implanted (*p* = 0.01). MLA at the level of the length was only slightly bigger in the group where regular DES was implanted, Fig. 2. Also, PV of the entire lesion and at the level of the bifurcation after stenting decreased in both groups; however, these changes were not statistically significant. On the contrary, PB changes were statistically significant; though they were not significant between both groups (Fig. 3). In general, LA significantly increased at all sites in both groups, but an increase in VA was found significant only at the window level in the DES group. As for PLA, there was a trend towards increasing at window at the window with DES. A schematic illustration of % CSA variation at regions of interest is shown in Fig. 4. Lastly, measurements of vessel, lumen and plaque CSA before and after stenting create an opportunity to identify mechanisms of the lumen enlargement at regions of interest. Overall, 2 mechanisms appeared to contribute to poststenting lumen increase, including vessel extension (stretch) and plaque re-distribution, Table 5.

**Table 4:** Baseline and final IVUS measurements comparing DES versus BiOSS groups

Variable	DES		BiOSS		*p* value	
	Pre (a)	Post (b)	Pre (c)	Post (d)	Pre (a vs. c)	Post (b vs. d)
MLA site						
LA, mm^2^	2.87 ± 0.78	6.08 ± 2.01	2.99 ± 0.82	6.49 ± 2.2	0.68	0.68
VA, mm^2^	14.79 ± 4.75	16.43 ± 4.95	17.37 ± 7.57	18.04 ± 8.03	0.26	0.5
PLA, mm^2^	11.88 ± 4.47	9.63 ± 3.71	14.37 ± 7.02	11.54 ± 6.25	0.24	0.3
PL site						
LA, mm^2^	4.78 ± 1.49	7.86 ± 2.08	3.89 ± 0.98	7.84 ± 1.99	0.06	0.97
VA, mm^2^	16.36 ± 3.77	18.01 ± 5.16	19.23 ± 6.79	20.91 ± 8.24	0.15	0.24
PLA, mm^2^	11.59 ± 3.79	10.47 ± 4.02	15.35 ± 6.48	13.08 ± 6.99	0.05	0.21
DL site						
LA, mm^2^	5.21 ± 3.18	7.46 ± 2.2	4.78 ± 2.18	6.44 ± 1.85	0.66	0.17
VA, mm^2^	14.25 ± 5.38	16.59 ± 4.61	13.2 ± 4.22	14.53 ± 4.86	0.54	0.23
PLA, mm^2^	9.06 ± 3.4	9.09 ± 3.71	8.45 ± 2.59	8.09 ± 3.67	0.57	0.45
Window area						
Window length, mm	2.31 ± 0.38	1.76 ± 0.52	2.09 ± 0.50	2.21 ± 0.37	0.79	0.01
LA, mm^2^	4.86 ± 2.44	7.63 ± 2.03	3.99 ± 1.19	6.52 ± 1.64	0.21	0.1
VA, mm^2^	13.89 ± 2.59	17.56 ± 5.22	13.71 ± 3.98	14.88 ± 4.31	0.88	0.12
PLA, mm^2^	9.06 ± 2.27	9.94 ± 4.32	9.72 ± 3.91	8.36 ± 3.99	0.56	0.29
Volumetric analysis						
PV, mm^3^	174.44 ± 49.88	153.13 ± 36.46	164.43 ± 62.35	149.42 ± 68.01	0.64	0.86
PB, %	64.2 ± 4.6	50.9 ± 4.6	60.8 ± 9.1	50.5 ± 7.9	0.21	0.86
PV at in-bifurcation segment, mm^3^	42.9 ± 14.09	35.53 ± 6.67	47.41 ± 19.35	40.35 ± 15.87	0.48	0.30
PB at in-bifurcation segment, %	66.5 ± 9.3	53.9 ± 8.8	72.1 ± 6.9	55.2 ± 9.2	0.07	0.71

Values are presented as mean ± standard deviation

*DL* distal limb, *LA* lumen area, *MLA* minimum lumen area, *PB* plaque burden, *PL* proximal limb, *PLA* plaque area, *PV* plaque volume, *VA* vessel area

**Table 5:** Mechanisms of post-stenting lumen enlargement at regions of interest

Variable	Group	PL (%)	Window (%)	DL (%)	Target stenosis (%)
Vessel extension	DES	54	130	98	51
	BiOSS	43	46	80	19
Plaque re-distribution	DES	36	−30	2	49
	BiOSS	57	54	20	81

## Discussion

The increasing experience in percutaneous coronary interventions, the continuous improvement of technical parameters of angioplasty materials and encouraging results obtained with new DES generations cause a broader selection of coronary stenoses and opening for lesions, which were considered not long ago as uninviting for percutaneous coronary interventions [8]. Undoubtedly, lesions located at coronary bifurcations are a great example of such stenoses and their rate is systematically increasing among catheterized patients [9].

It has been proven that DES based on the classical construction, where basic elements are the same along a stent, are biased by many limitations. The most important is maximal cell`s size [10, 11]. As a result that construction particularly in case of not optimal stent implantation may predispose not only to periprocedural complications like side branch closure, intra-stent thrombosis, but also to long-term complications, such as restenosis and late or very late stent thrombosis [12, 13]. These facts are good reasons to search for a stent a`priori designed for a coronary bifurcation (DBS–dedicated bifurcation stent). There are many publications regarding those devices and their number systematically increases [14–18]. Among four DBS`s types, the most favored seems to be this designed for the parent vessel treatment and simultaneous protection and access to the side branch [19], given that the ESC guidelines strongly recommends provisional side branch stenting taking into consideration not only immediate but also long-term results of this strategy [8].

The BiOSS stent belongs to the above mentioned type of DBS. Its construction have rose the question on whether 1.2 mm long intermediate zone of that stent appears as the weakest part predisposing to restenosis and intra-stent thrombosis. Recently published 3-month results [1] and already known 12-months results [20] of BiOSS Expert Registry deny those assumptions. However, we decided to assess whether the implantation of the BiOSS stent impacts mechanisms involved in lumen enlargement and consequently, side branch compromise, in comparison with classical provisional T-stenting strategy with DES. The results of the QCA analysis performed in the studied population were not surprising, bearing in mind the well-known weakness of this method [9]. Our data unequivocally indicate that final results achieved in both groups (MLD, diameter stenosis) did not differ significantly. However, it deserves to note that in the group where the BiOSS stent was used, final diameter stenosis at side branch significantly decreased on the contrary to the other group. This finding was confirmed in 12 months analysis of BiOSS Expert Registry as well [20]. IVUS was chosen as the tool for the comparative assessment. It is known that this invasive diagnostic method gives opportunity not only to analyze the enlargement of the vessel and lumen, but also changes in vessel and atherosclerotic plaque areas [9]. According to the assumption, more definitive information was obtained by the analysis of IVUS recordings. It must be underlined that qualitative and quantitative analyses did not show significant preprocedural differences between both groups. The analysis of classical quantitative ultrasonic parameters allowed to ascertain that both classic DES as well as BiOSS stenting enable to obtain the comparable increase of lumen in most stenosed parent vessels (main vessel + main branch). Simultaneously, the BiOSS stent construction (of course in case of proper implantation) provides a better access to side branch in comparison to the classic DES. It was proven by significantly bigger window length in the BiOSS group—a parameter which represents the access to the side branch.

It is known from the literature that the mechanism of the stent expansion is a combination of the vessel stretch and the plaque “reduction” [21, 22]. However, more detailed studies analysis have shown that the last mechanism is more complex [23, 24]. It consists of the axial redistribution of atheromatous plaque [25, 26] and plaque compression [23] rather than embolization [27]. Previous IVUS studies have demonstrated that in non-calcific lesions, the mechanisms of the lumen enlargement after stenting (direct implantation or after predilatation) are significantly influenced by atherosclerotic remodeling, plaque eccentricity and plaque composition [21]. Birgelen et al. showed that a proper remodeling pattern of coronary lesions has significant impact on mechanisms of lumen enlargement during stent deployment. Lesions with positive remodeling showed more plaque extrusion into distal reference and less stent-induced vessel stretch than those with negative remodeling [22]. They also found that marked plaque extrusion occurs only in lesions with calcium arc <120° within the vessel circumference. In both groups, vessel and lumen areas increased equally, while the plaque area decreased after stent deployment [24]. Algowhary et al. [25] showed axial redistribution of atheromatous plaque along the segment and proximal and distal reference segments. Prati et al. [27] found that the decrease in the plaque area during stenting predicts CK-MB release suggesting a high association between stenting and plaque embolization in patients with unstable angina pectoris. Also, Maehara et al. [26] found that plaque re-distribution, not compression, as a result of stent expansion translates disease accumulation from the mid-stent zone to the distal stent zone, given that after balloon postdilatation, the additional lumen gain is proportional to more plaque redistribution rather than vessel expansion. According to Dudek et al. [23] stenting causes vessel expansion to accommodate the plaque mass pressed by the stent and longitudinal plaque redistribution along the stented segment with plaque shifting to the proximal and distal reference segments.

Nonetheless, there are some differences between cited above papers and our work. Firstly, we studied bifurcation lesions in patients with stable coronary artery disease and secondly, we decided to perform comparative analysis of two stents different in design. The fact that there were not significant differences in terms of preprocedural IVUS parameters (qualitative and quantitative) increases reliability of the above mentioned analysis. It is worthy to be stressed that precise analysis of changes in vessel, lumen and plaque areas along the lesion to be stented in key places (proximal and distal limbs, target stenosis, window length) of bifurcation allows to define in detail the influence of the BiOSS construction on mechanisms of lumen enlargement. To our knowledge this is first paper on mechanisms of lumen enlargement after coronary stenting of bifurcation lesions. It is very interesting that we did not find uniform operating mechanisms not only between two compared stents but also within both groups taking into account measurements along the lesion. And so, in the BiOSS group at the level of target stenosis, the greater degree of lumen enlargement was achieved due to plaque “reduction” versus vessel expansion (81 vs. 19 %). On the contrary, in the DES group, those two mechanisms played a similar role (50 vs. 50 %). Completely different relations were found at the level of distal limb where vessel expansion was the superior mechanism over plaque reduction (80 vs. 20 % in BiOSS; 98 vs. 2 % in DES; respectively). This small PLA decrease at the level of distal limb in the DES group seems to be in association with axial plaque redistribution, especially that in the adjacent site—window region. In the same group (DES), we found PLA increase in the contrary to the BiOSS group, where two mechanisms were quite well balanced (43 % vessel expansion vs. 57 % plaque reduction). Those findings confirm that the construction of the classical stent does not take into consideration vessel tapering in bifurcation lesions and results in carina and plaque shift—the main mechanisms of side branch compromise. Furthermore, the analysis of plaque, lumen and vessel areas at the level of proximal limb showed that the mechanisms were similar in both groups. However, a trend to a bigger vessel lumen increase with simultaneous plaque “reduction” at the site of proximal limb in the BiOSS group confirmed that the construction of that stent assures the realization of the proximal optimization technique (POT) which is strongly recommended by European Bifurcation Club [28]. If add proofs for smaller changes in vessel and lumen areas at the level of the distal limb after BiOSS stent implantation and, not surprisingly, smaller residual stenosis at side branch ostium in the group where that stent was used. These findings confirm, in an indirect manner, that the BiOSS stent construction limits carina and plaque shift towards side branch, which are two major factors responsible for side branch compromise. As a consequence, such design affects less the in-bifurcation segment; this is expressed by significantly smaller VA increase, negligible differences in LA increase and PLA reduction. These observations prove that construction of the BiOSS stent enables more physiological fitting for the bifurcation anatomy. Moreover, it allows to believe that the principle of “less injury, less vessel response (less neointimal proliferation)” may translate into very good clinical results [29, 30]. Also, it must be stressed that postprocedure analysis revealed that there was main branch stent oversizing in DES group, but not in BIOSS group, and this might be an additional factor responsible for the final results of our study.

Finally, analysis of PV changes, which were equal for entire lesion as well as for bifurcation specific segments, proves that the implantation of both types of stents leads to its insignificant reduction (Table 4). However, it should be stressed that PB parameter, obtained by volumetric analysis of residual plaque area in relation to VA within the segment of interest, underwent significant reduction in both studied groups. The value of this parameter, which was around 50 % plus the presence of a potent antiproliferative drug, seems to be an additional argument for at least similar long-term results for classic DES versus BiOSS stents [31].

## Conclusions

Our results suggest different mechanisms of lumen enlargement in coronary bifurcation lesions treated by percutaneous coronary interventions with provisional approach with conventional DES versus the BiOSS DBS. Overall, there comparable luminal gain, but the BiOSS stent was associated with less luminal compromise and plaque re-distribution at the level of the side-branch in-flow at the in-bifurcation segment.

## Abbreviations

CSA: Cross-sectional area
EEM: External elastic membrane
IVUS: Intravascular ultrasound
LA: Lumen area
MLA: Minimum lumen area
MLD: Minimum lumen diameter
PB: Plaque burden
PLA: Plaque area
PV: Plaque volume
VA: Vessel area
